# Real-time, selective, and low-cost detection of trace level SARS-CoV-2 spike-protein for cold-chain food quarantine

**DOI:** 10.1038/s41538-021-00094-3

**Published:** 2021-06-01

**Authors:** Jian Zhang, Xin Fang, Yu Mao, Haochen Qi, Jayne Wu, Xiaoru Liu, Fangshuo You, Wenci Zhao, Ying Chen, Lei Zheng

**Affiliations:** 1 School of Electronic Science and Applied Physics, Hefei University of Technology, Hefei, China; 2 School of Food and Biological Engineering, Hefei University of Technology, Hefei, China; 3 Department of Electrical Engineering and Computer Science, The University of Tennessee, Knoxville, TN USA; 4 Agro-product Safety Research Centre, Chinese Academy of Inspection and Quarantine, Beijing, China

**Keywords:** Assay systems, Biosensors

## Abstract

Due to the friendly temperature for virus survival, SARS-CoV-2 is frequently found in cold-chain foods, posing a serious threat to public health. Utilizing an interdigitated microelectrode chip modified with an antibody probe and integrating dielectrophoresis enrichment with interfacial capacitance sensing, a strategy is presented for the detection of trace level spike-protein from SARS-CoV-2. It achieves a limit of detection as low as 2.29 × 10^−6^ ng/mL in 20 s, with a wide linear range of 10^−5^–10^−1^ ng/mL and a selectivity of 234:1. The cost for a single test can be controlled to ~1 dollar. This strategy provides a competitive solution for real-time, sensitive, selective, and large-scale application in cold-chain food quarantine.

## Introduction

The contamination of pathogens in food and the environment has always been a serious problem worldwide for food safety, entry-exit quarantine, epidemic prevention, and public health management. Although viral epidemics are less reported for food safety compared with bacteria^1^, their transmissibility is not weak at all. More typical examples have been showing their destructive power, such as large outbreaks of hepatitis A^2,3^ and norovirus infection^4,5^. As expected, these viruses are detected in various foods as well as water^1,6,7^, which exacerbates the spread of the disease. Therefore, the viral contamination detection in food poses a more great challenge in the recent decade^8^.

Since winter 2019, the outbreak of a so-called coronavirus disease 19 (COVID-19) has become a global pandemic, with the culprit being a new SARS coronavirus (SARS-CoV-2)^9^. Although there is not yet a direct evidence to indicate the foodborne characteristic of SARS-CoV-2, food contamination of SARS-CoV-2 has presented a risk for virus spreading, which attracts considerable attention in many countries^10–12^. SARS-CoV-2 virions adhering on the solid surface are reported to be stable, with a viability up to longer than 72 h (on plastic)^13^. As a matter of fact, besides the wet markets providing a breeding ground for the virus^14^, positive identification of SARS-CoV-2 on cold-chain foods are frequently reported in China recently, including the confirmed SARS-CoV-2 virions on plastic packing. Due to the below 0 °C temperature during the cold-chain transportation, the viruses can have a longer survival time than in room-temperature environment. On the other hand, the virus concentration may be of trace level, while a large number of samples need to be tested. Therefore, sensitive, fast, and low-cost SARS-CoV-2 detection is urgently needed for cold-chain foods.

For virus detection in food, there are mainly two types of techniques, culture/counting methods and polymerase chain reaction (PCR)-based methods^7,8^. Because the culture process is very time-consuming, PCR-based methods have become a mainstream approach^8,15^. Numerous works on various virus detection by PCR or reverse transcription-PCR (RT-PCR) in food have been reported, including hepatitis A^15–17^, hepatitis E^18^, and norovirus^15–17^. Although PCR techniques have been developed over the past 30 years, the operation is still complicated, and the required turnaround time cannot be shorter than several hours^15^. Based on the good specificity and versatile sensing mechanisms, bio-probe-based sensors for virus detection are becoming more practicable in recent years as promising approaches^19–21^. Also, due to the high specificity, reproducibility, and stability, antibody-based detections of causative agents are continually adopted in food safety detection^22,23^.

A SARS-CoV-2 virion binds directly with its receptor through spike (S-) protein on its surface, and the subunit of S1 serves as the receptor-binding domain^24–26^. As a result, S-protein (S1 subunit) is selected as a preferred biomarker for SARS-CoV-2 detection^27–29^. Because the S-protein existing on the periphery of the virion is exposed on the food surface independent of body infection and viral assembly, at a quantity more than viral RNA itself, it can act as a good biomarker for food contamination of SARS-CoV-2. For specific recognition of certain antigens, the antibody is considered as a promising probe due to the usually high affinity between the antigen and antibody^30^.

Here, a method for trace S-protein (S1 subunit) detection associated with cold-chain food is developed for real-time SARS-CoV-2 contamination screening. An anti-SARS-CoV-2 S-protein antibody is immobilized on a low-cost interdigitated microelectrode (IDME) chip. Then, an alternating current (AC) signal is applied to the IDME to induce a dielectrophoresis force on the protein particles. Thus, the S-proteins are rapidly driven toward the IDME surface in the nanofluid and captured by the antibodies. Utilizing the electrical double layer capacitance at the IDME surface as an ultra-sensitive indicator, the limit of detection (LOD) reaches 10^−6^ ng/mL, while the time from sample to result is as short as 20 s due to effective target enrichment embedded into the detection process. Meanwhile, the detection is specific against the interferences, demonstrating a satisfactory selectivity in S-protein recognition. This report constructs a cost-effective immunosensor as well as a simple detection strategy for real-time, selective, and large-scale screening of SARS-CoV-2 for cold-chain food quarantine.

## Results and discussion

### Characterization of sensor preparation

**Fig. 1:**
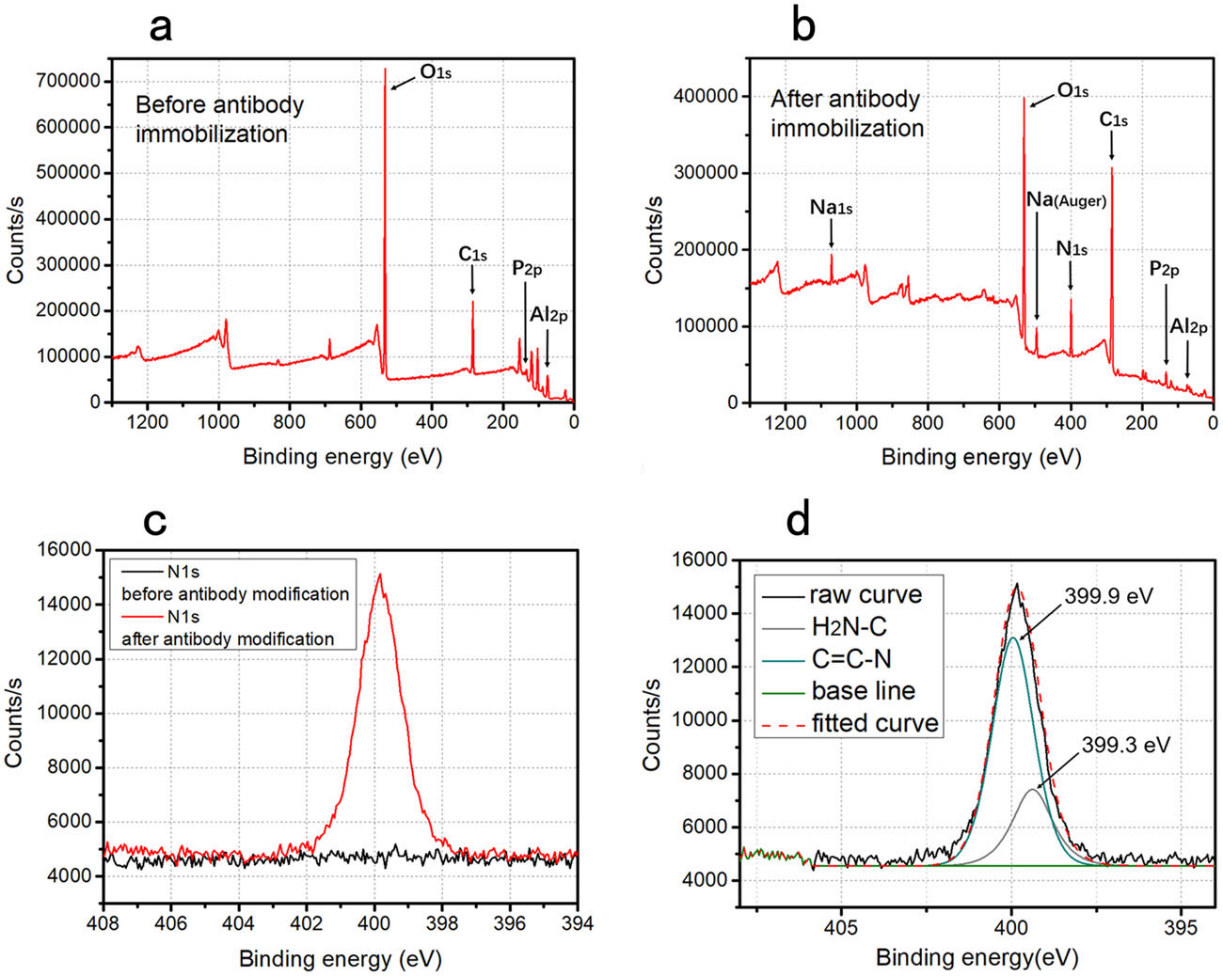
Characterization for sensor’s functionalization by XPS spectrums. Full spectrum on **a** thoroughly cleaned IDME surface before antibody modification and **b** functionalized IDME surface after antibody modification. **c** High-resolution spectrums of nitrogen (N1s) before and after IDME modification. **d** The peak differentiating and imitating of N1s.

For sensor preparation, the functionalization should be verified before its application. In this work, the commercial IDME is fabricated by sputtering aluminum on the substrate, so Al (Al2p) should be first verified. Al (Al2p) is dominant at the IDME surface before functionalization (Fig. 1a), demonstrating the surface Al element and the surface cleanliness, but it is difficult to find after the antibody immobilization (Fig. 1b). According to original data, the peak area of Al2p decreases from 17,544 to 1916 cps⋅eV, which is due to good antibody coverage on the IDME surface. Antibody coverage is also confirmed by the appearance of nitrogen (N1s) characteristic peak (Fig. 1c), which is a characteristic element from the antibody. According to peak differentiating and imitating (Fig. 1d), the formula of N1s contains (H2N-C) and (C=N)^31^, which is abundant in the antibody. Sodium (Na1s) and phosphorus (P2p) (Fig. 1b) are accordant with the characteristic peaks from Na_2_HPO_4_ and NaCl, the components of phosphate-buffered saline (PBS) solvent. The presence of P2p at the bare IDME (Fig. 1a) could be residues of phosphoric acid from Al electrode etching during chip fabrication. In conclusion, the X-ray photoelectron spectroscopy (XPS) spectrums validate a successful surface functionalization by the antibody.

**Fig. 2:**
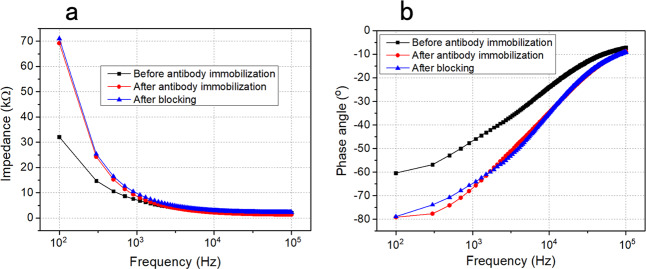
Characterization for sensor’s functionalization by Bode plots. **a** Impedance and **b** phase angle from 10^2^ to 10^5^ Hz to describe the IDME surface modification.

In addition, Bode plot of impedance and phase angle from 10^2^ to 10^5^ Hz is acquired to reflect the change on the IDME surface during functionalization by an electrical method. The impedance distinctly increases after antibody modification, but changes little after blocking (Fig. 2a). Because the covered antibody layer makes the dielectric layer thicker on the IDME surface, causing a smaller conductivity than that of a single electric double layer (EDL), the increase of impedance is easy to understand. The reason why the lactalbumin blocker changes little of the impedance is the smaller lactalbumin not making the dielectric layer thicker anymore. In the phase angle spectrum (Fig. 2b), the lag of phase angle after antibody modification verifies the interfacial capacitance. Also, the blocking has little effect on the phase angle curve because of its weak influence on the capacitance.

### Dose response from S-protein in PBS buffer

The calibration for the IDME immunosensor should be first performed before its application. The S-protein is tested with ten-fold increase from 10^−5^ to 10^−1^ ng/mL in 0.1× PBS solution. Here, the upper-limit concentration of 10^−1^ ng/mL is determined by observing the saturation phenomenon^32,33^ at 1 ng/mL (illustrated in Fig. S1a, b in Supplementary Material).

**Fig. 3:**
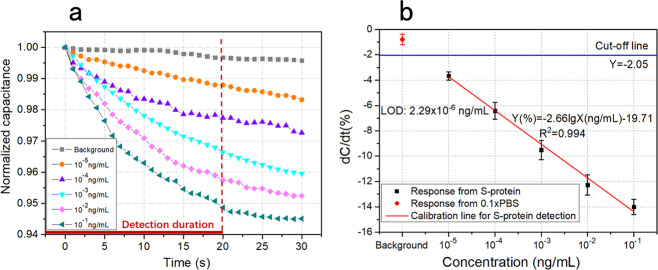
Response acquired from the immunosenors. **a** The typical transient capacitance normalized by its initial value changing with time. Five concentrations of S-protein (from 10^−5^ to 10^−1^ ng/mL) as well as the background of 0.1× PBS are continuously detected for 30 s. **b** Dose response and its calibration. The triplicate data are acquired from three freshly prepared sensors, and the error bar represents the standard deviation.

The typical transient curves of normalized capacitance vs. time are acquired from the sensors (Fig. 3a). The curves can be clearly differentiated between different target concentrations and exhibit increasingly downward slopes with the increase of S-protein concentration in 0.1× PBS. This indicates the sensor’s primary performance of output monotonicity. The linearity of the transient curves becomes worse after 10 s when the concentration is high. Therefore, the response time of this sensor is chosen to be 20 s.

For calibration using dose response (Fig. 3b), the response of capacitance is normalized by the initial one, and d*C*/d*t* represents the change of normalized capacitance in 1 min duration, which is found by least-squares linear regression method. The calibration curve, *Y* (%) = −2.66lg *X* (ng/mL), demonstrates an excellent semi-log linear relationship between the target concentration and the response from 10^−5^ ng/mL until 10^−1^ ng/mL, with a correlation coefficient (*R*^2^) of 0.994. With the cut-off line (*Y* = −2.05%) defined as a response value three standard deviations from the background, the LOD is calculated to be 2.29 × 10^−6^ ng/mL plugging the cut-off value into the calibration equation. This LOD is at an ultra-low level. The dose response as well as the calibration demonstrates a quantitative performance of the sensor, with a wide linear range.

### Selectivity of S-protein against the interferences

For a qualified sensor, selectivity for the target is the most important performance. In this work, the acquired effective response is verified in three ways: (1) comparing the response from functionalized sensors with that from dummy sensors when S-protein is the target of the test, (2) comparing the response from S-protein with that from the background of 0.1× PBS, and (3) comparing the response from S-protein with that from the interferences. Here, the dummy sensor is defined for an IDME chip without antibody functionalization to verify the probe’s effectiveness.

**Fig. 4:**
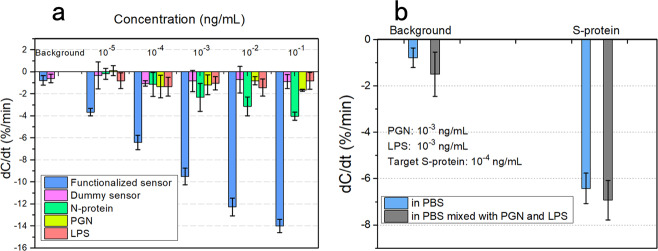
Specificity verification of the immunosenors. **a** Specificity verification using different types of sensors and different analytes. The background is first tested to verify the blank control and the sensor blocking effect. Functionalized sensors are compared with dummy sensors (also blocked with lactalbumin), to characterize the antibody functionalization. The control group contains nucleocapsid (N-) protein, peptidoglycan (PGN), and lipopolysaccharide (LPS). **b** Specificity verification in the hybrid medium. A hybrid medium is constructed by mixing PGN and LPS in 0.1× PBS solution. The PGN and LPS concentrations in 0.1× PBS are both 10^−3^ ng/mL, and the spiked S-protein is at 10^−4^ ng/mL. In both (**a**, **b**), the error bar represents the standard deviation from three tests of the same sample using different sensors of the same batch.

According to the comparison result (Fig. 4a), the response from the background is within 1%, the dummy sensors keep unresponsive, and the two interference molecules of peptidoglycan (PGN) and lipopolysaccharide (LPS) both cause an unobvious response. The biggest nontarget response is from the SARS-CoV-2 nucleocapsid (N-) protein, especially when its concentration is high. The maximum of −4.04% from N-protein at 10^−1^ ng/mL is equivalent to the response from S-protein at 4.27 × 10^−4^ ng/mL calculated using the calibration line, then the selectivity ratio of this sensor is calculated to be 234:1 (10^−1^ ng/mL:4.27 × 10^−4^ ng/mL).

To demonstrate the capability of target recognition in hybrid matrices, S-protein detection is also performed in 0.1× PBS containing PGN and LPS, both at a concentration of 10^−3^ mg/mL, which most possibly exist at the food surface dissociated from various bacteria. In this test, the spiked S-protein is 10^−4^ ng/mL. Although the concentrations of nontarget molecules are 10-fold higher than that of S-protein, the response is only a bit larger than that in PBS without impurities (Fig. 4b), and still within an acceptable tolerance from the calibration curve. The response is ~107.94% of the calibrated concentration, meeting the requirement of practical quantitative detection in hybrid matrices.

### Detection of S-protein in practical media

After verification of the sensor performance as well as the test strategy, we have applied this approach in practical media for trace S-protein detection. Three types of media associated with cold-chain food are modeled: melted tap water from the ice for cold-chain transportation, extracts from different fresh seafood surfaces, and soaking liquid from a packing bag for frozen food. Because the food samples confirmed to be contaminated by SARS-CoV-2 are extremely difficult to obtain, different matrices spiked with S-protein are detected.

**Fig. 5:**
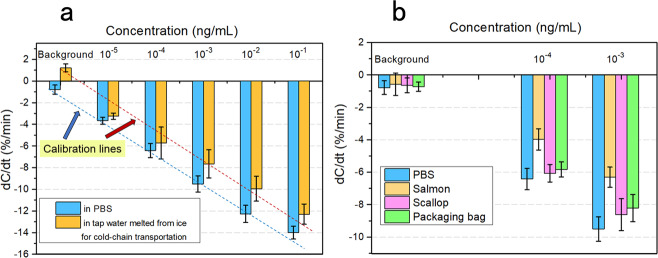
S-protein detection in practical samples. **a** Dose response from S-protein in melted tap water from the ice for cold-chain transportation. Five concentrations from 10^−5^ to 10^−1^ ng/mL are spiked and detected in the tap water, and the calibration is performed as a red dashed line. The blue dashed line as a standard calibration in PBS is provided for comparison. **b** S-protein detection in three samples associated with cold-chain food. Leaching solutions are obtained from salmon, scallop, and packing bag for beef, all having two theoretical concentrations (10^−4^ and 10^−3^ ng/mL). The corresponding backgrounds are as a quality control for the sensors before application. The response in 0.1× PBS is provided as a reference. In Fig. 5, each error bar representing standard deviation is obtained using the responses from the same sample tested with three sensors.

When S-protein detection in the tap water is performed (Fig. 5a), the spiked concentration is from 10^−5^ to 10^−1^ ng/mL. For comparison, the detection result from spiked 0.1× PBS is provided together and the response from the tap water is lower than from PBS. The background of tap water is above zero, which may be caused by very few large particles such as bacteria and dust in the solution. If the adsorbed particles are too few in number and the particle size is large (up to nm or μm), the interfacial capacitance will increase as illustrated in Supplementary Material (Fig. S2). In Fig. 5a, although the baseline of the tap water is >0, the variation tendency of response keeps in good agreement with that from spiked PBS, showing an expected dose relationship. For practical application, the response will reflect the real S-protein concentration after a simple baseline calibration.

Other detections for S-protein associated with cold-chain food are also performed with three types of materials, i.e., salmon, scallop, and a packing bag for frozen beef (Fig. 5b). Here, two concentrations of 10^−4^ and 10^−3^ ng/mL are obtained by spiking, incubation, and dilution steps as introduced in the “Methods” section. To get rid of the impurity such as lipid particles, centrifugation is also executed before final detection. Four backgrounds all show negligible response ~1% of d*C*/d*t*, indicating a qualified sensor preparation to block nonspecific adsorption. The target S-protein in various matrices can be distinctly recognized at both concentrations, although the responses are smaller than that from PBS solution. In these results, the smallest response is from the salmon. Because the salmon sample has been processed in strips, and the lipid is rich at the surface, there are many organic molecules and particles dispersed and suspended in the turbid solution. Although high-speed centrifugation has made the background unresponsive, the positive response may be inhibited to some degree. For further practical application with these complex samples, a new calibration can be made for the accurate quantitative determination of SARS-CoV-2 contaminant. By contrast, the surface of the scallop and packing bag is much smoother and denser, so that the interference is much less, and the S-protein can be more completely dispersed in the PBS solution. As a result, the responses from these two samples are more notable. According to these results, S-protein in different cold-chain food samples can be successfully detected by our sensing method.

## Discussion

As a key target for SARS-CoV-2 recognition, S-protein shows a great value for the virus recognition besides drug and vaccine development. In this work, a strategy for real-time, selective, and low-cost detection of S-protein from SARS-CoV-2 is presented based on a low-cost commercial IDME-based sensor combined with interfacial capacitance-sensing method. An anti-SARS-CoV-2 S-protein antibody is employed as a bio-probe immobilized on the IDME surface to specifically recognize the trace S-protein in cold-chain food-associated samples.

Due to the effective utilization of dielectrophoresis (DEP) force, S-protein particles are attracted towards the sensor’s surface and captured by the antibodies in 20 s. Also, the response acquisition is completely integrated into the target enrichment process, thus the response time is shortened to 20 s, a duration meeting the real-time detection demand on site. Besides, the LOD of this detection reaches an ultra-low level, i.e., 10^−6^ ng/mL. Meanwhile, the linear range of 10^−5^–10^−1^ ng/mL is extremely wide covering the possible range of S-protein concentration at the food surface. Even when testing a food sample with a complex surface such as a salmon strip, the presence and concentration of S-protein can be easily determined. In fact, cold-chain foods are mostly contaminated with SARS-CoV-2 virions by the workers carrying the virus in food-processing factories. Because virus replication cannot occur at the food surface, the level of virus concentration should be very low, which poses a great challenge to the traditional detection methods. As such, the ultra-low LOD of the developed strategy shows an immense advantage in trace virus marker detection.

Another merit of this strategy is the high specificity. The selectivity for S-protein against the interference is calculated to be 234:1. Not only common organic molecules but also various matrices as backgrounds are verified to be nonresponsive, with known or unknown components. Therefore, the sensor has demonstrated the required specificity to identify the S-proteins from practical samples with complex backgrounds.

The cost for a single sensor device is estimated to be ~1 dollar. As a result, this sensor is designed to be a disposable chip. Meanwhile, the impedance analyzer for capacitance detection is also inexpensive and lightweight. As discussed previously, the detection operation as well as the pretreatment is simple, only including dilution and centrifugation steps. Therefore, this sensor can be operated by a layperson. Based on the above characteristics, the platform and method for S-protein detection provide a promising solution for large-scale applications in cold-chain food quarantine requiring a quick response, low LOD, high specificity, user-friendly operation, and low cost as well.

## Methods

### Target enrichment and sensing mechanism

For trace particle detection in liquid, the preconcentration of target particles is always crucial to a successful test^34–36^. Compared with most techniques for preconcentration needing an extra incubation or equipment, AC electrokinetic (ACEK) effects can manipulate nanoparticles efficiently to realize target enrichment rapidly without extra processes or devices^37–39^. For relatively large molecules such as proteins, DEP force, an important ACEK effect, has been demonstrated as a dominant force applied on the particles^40,41^. In this work, an IDME chip is used as a physical device to induce the DEP effect. DEP force can be expressed as a function of electric field (Equation S1 in Supplementary Material), affected by the electric field gradients in the liquid. The voltage applied to the IDME has a positive correlation to the DEP force. To illustrate the space distributions of electric potential and the induced DEP in solution, their simulation results are provided (in Fig. S3), according to which the potential and velocity field are described under the test condition, and the directional movement of S-proteins towards the IDME surface is simulated. Therefore, the nanofluidic manipulation and enrichment of S-protein can be expected.

When an electrode is immersed in a solution, EDL will appear due to the accumulated charges at the electrode surface and the layer of induced counter ions above the surface^42,43^. The layer of antibody and lactoalbumin molecules at the IDME surface plus the EDL forms the initial dielectric layer of the interfacial capacitance (as shown in Fig. S4). When S-proteins are captured by the antibodies, the dielectric layer becomes thicker, and as a result, the interfacial capacitance becomes smaller (as deduced in Eq. S2). The change of normalized capacitance per minute, i.e., d*C*/d*t* (%/min), directly reflects the S-protein adsorption level, by which the S-protein concentration in the solution can be indicated. Using an IDME of micron-scale, d*C*/d*t* is a competitive parameter to reflect the tiny change at the electrode interface^38,44^. Therefore, ultra-trace S-protein detection can be expected. In practice, the lab-prepared sensors may have inconsistency due to different effective electrode surfaces and total recognition sites. Here, the normalization by the initial capacitance (as described in Eq. S2) has no relation with the initial electrode surface, and thus can minimize the deviation between the response from different sensors.

### Materials and reagents

The S-protein (S1 subunit) is purchased from Cellregen (Beijing) Life Science and Technology Co., Ltd, which is recombinant and expressed by the prokaryotic system with the host of *Escherichia coli*. The molecular weight of this protein is 75.3 kDa, with a purity >90%. The sodium dodecyl sulfate-polyacrylamide gel electrophoresis result is provided in Fig. S5a, and the amino acid sequence is shown in Fig. S5b. The anti-SARS-CoV-2 spike-protein S1 (mouse monoclonal IgG) is purchased from Anygo Technology Co., Ltd, China, with a purity >95%. The recombinant N-protein is also provided by Cellregen (Beijing) Life Science and Technology Co., Ltd, and the LPS (L2880) is ordered from Sigma-Aldrich Co., Ltd. The PGN is bought from Nanjing Duly Biotechnology Co., Ltd, China. The IDME chips are modified based on commercially available surface acoustic wave (SAW) chips (AVX Corps’ Kyocera 433K).

### Sensor preparation and test protocol

The SAW chip is packaged by a ceramics chamber with an outer size of 5 × 3.5 mm^2^ (as shown in Fig. S6a). The chamber is enclosed by a metal cover. Inside the chamber, an aluminum IDME structure is fabricated on a ceramic substrate (as shown in Fig. S6b), the widths of the finger and gap of the IDME are 2 and 1.5 μm, respectively. For sensor preparation, the metal cover is first removed, and the chip is thoroughly cleaned by soaking in acetone for 25 min, soaking in isopropyl alcohol for 2 min, and rinsing with purified water for 10 s. Then, the chip is treated with ozone for 30 min to improve the hydrophilia of the electrode surface. After that, 10 μL antibody (10 μg/mL in 0.05× PBS) is added into the chamber and incubated for 24 h in a humidor. The final step is blocking for 3.5 h using lactoalbumin (100 μg/mL in 0.05× PBS) to cover the unfunctionalized IDME area.

The functionalized sensor is first connected to an impedance analyzer (TH2829C, Tonghui Electronic Co., Ltd) (as shown in Fig. S7a). After the sample is dropped into the sensor’s chamber, an AC signal is applied for a selected duration of 20 s.

The test procedures mainly include incubation, extraction, and centrifugation steps (as shown in Fig. S7b). Before measurement, all the practical samples should be pretreated. The conductivity of raw tap water (melted from the ice for cold-chain transportation) is tested to be 0.012 S/m. Then, the raw tap water is 1:1 mixed with 0.19× PBS to obtain a mixture with the conductivity of 0.141 S/m, which is as same as that of 0.1× PBS. During all the detection in this work, the solution conductivity is kept to be 0.141 S/m, which makes an equal effect on DEP by solution conductivity^28,29^. Then S-protein is added in this mixed background also called as “tap water”. For cold-chain food-associated samples (salmon, scallop, and a packing bag for frozen beef), a small piece of a certain sample of ~8×8 mm^2^ is first cut-off, and 10 μL S-protein of 1 ng/mL is spread over it. After the solid sample is incubated for 12 h in a closed tube at 4 °C, 990 μL of 0.1× PBS is added into the tube. Then, the solution is sufficiently agitated and centrifuged at 3000 r.p.m. for 10 min. The supernatant from this mixture with a theoretical S-protein concentration of 10^−2^ ng/mL is ready for further dilution to obtain the final samples with theoretical concentrations of 10^−3^ and 10^−4^ ng/mL. During pretreatment of the salmon slice, an additional centrifugation at 5000 r.p.m. for 10 min is necessary to remove the abundant interference, and the floating lipid layer should be excluded when the supernatant is collected. To obtain the corresponding backgrounds for these practical samples, a similar process is performed except for the S-protein spreading and incubation steps.

The frequency of the AC signal is set to be 100 kHz according to a previous study^28,41^, and the voltage is optimized to be 100 mV according to the optimization (described in Fig. S8). Based on the measured capacitance, d*C*/d*t* can be calculated and analyzed. To demonstrate the sensor’s repeatability and consistency, all the data are presented with error bars, which represent the standard deviation obtained from three sensors of the same batch.

### Reporting summary

Further information on research design is available in the Nature Research Reporting Summary linked to this article.

## Supplementary information

Supplementary Information

Reporting Summary

## Data Availability

The authors declare that the data supporting the findings of this study are presented within the manuscript and the supplementary files. Additional data that support the findings of this study are available from the corresponding authors upon reasonable request.
