# Supplementary material for: Real-time, selective, and low-cost detection of trace level SARS-CoV-2 spike-protein for cold-chain food quarantine
Source: NPJ Sci Food. 2021 Jun 1;5:12. doi: 10.1038/s41538-021-00094-3 (PMC8357935; doi:10.1038/s41538-021-00094-3)
Supplement: Supplementary file 1 — Supplementary Information [file 41538_2021_94_MOESM1_ESM.pdf]

Supplementary Material

Real-time, selective and low-cost detection of trace level SARS-CoV-2 spike-protein for cold-chain food quarantine

Jian Zhang[1,2], Xin Fang[1], Yu Mao[2], Haochen Qi[1]✉, Jayne Wu[3]✉, Xiaoru Liu[1], Fangshuo You[1], Wenci Zhao[1], Ying Chen[4], Lei Zheng[2]✉

1 School of Electronic Science and Applied Physics, Hefei University of Technology, Hefei 230009, China.
2 School of Food and Biological Engineering, Hefei University of Technology, Hefei 230009, China.
3 Department of Electrical Engineering and Computer Science, The University of Tennessee, Knoxville, TN 37996, USA.
4 Agro-product Safety Research Centre, Chinese Academy of Inspection and Quarantine, Beijing 100123, China.

✉ Email: ammyqi@hfut.edu.cn (H. Qi), jaynewu@utk.edu (J. Wu), lei.zheng@aliyun.com (L. Zheng)

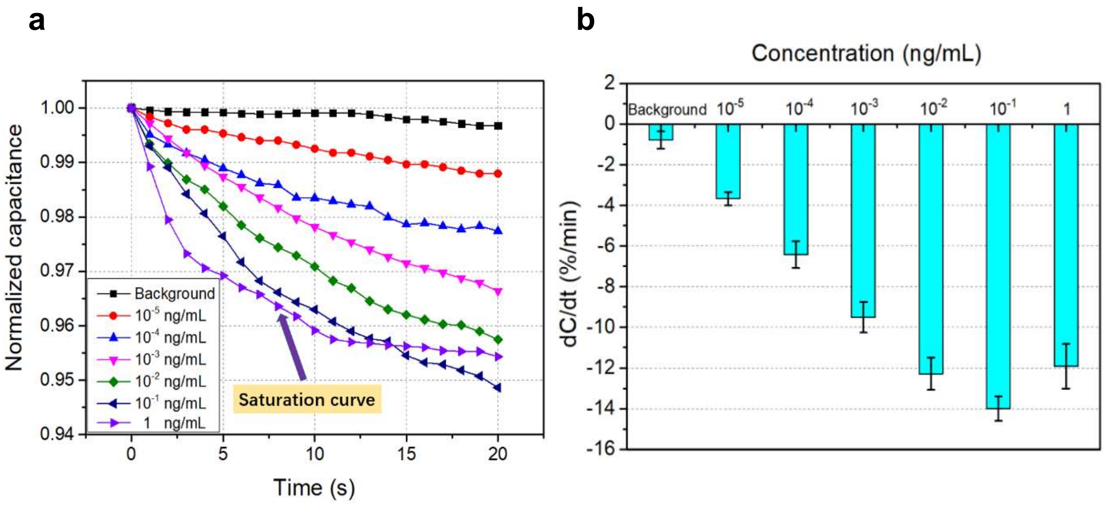

Fig. S1 Response saturation at a high S-protein concentration. a Transient curve of normalized capacitance change with time. The capacitance is normalized with respect to its initial value at t=0. The highest s-protein concentration is 1 ng/mL. An obvious leveling off of capacitance change occurs at this concentration, indicating adsorption saturation at the IDME surface. **b** The response of dC/dt from S-protein of (10^{-5} ~ 1) ng/mL. Every response is averaged from three sensors, with its standard deviation. Also, a decrease of the response (absolute value) is observed at 1 ng/mL, indicating the frequent appearance of target adsorption saturation.

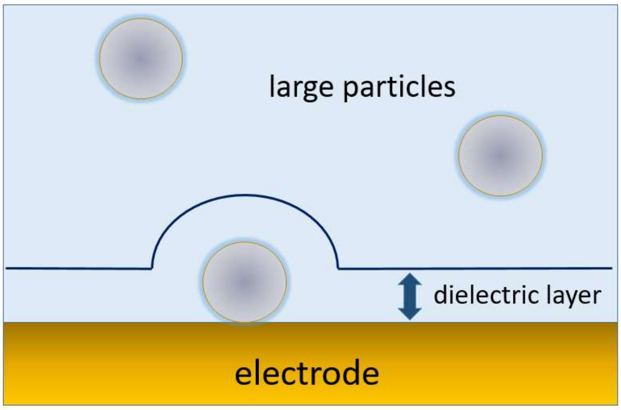

Fig. S2 Dielectric layer change caused by sparse and large particle adsorption on electrode surface. The spheres represent some “large” contaminants such as dust particles in melt-tap-water for ice-making in cold-chain transportation. The grain size might be from nano- to micro-meter, so that the influence can be not ignored. As illustrated in this figure, the adsorbed particles will bend the contour of EDL. When the particle count is not large enough to fill the sites on IDME surface, the dielectric layer surface area will increase, leading to an increase of the interfacial capacitance.

Eq. S1:

$$F_{DEP} = 2\pi\varepsilon_m r^3 \, \mathrm{Re}[K(\omega)] \nabla |E_{rms}|^2 \qquad \text{Eq. S1}$$

where ε_m is the relative permittivity of suspension medium, r is the particle radius. $\mathrm{Re}[K(\omega)]$ is the real part of Clausius-Mossotti factor and $K(\omega)$ is Clausius-Mossitti factor given by

$$K(\omega) = \frac{\varepsilon_p^* - \varepsilon_m^*}{\varepsilon_p^* + 2\varepsilon_m^*}$$

where ε^*_m is the composite permittivity of the suspension medium, ε^*_p is the composite permittivity of the particle, and ω is the electric field angular frequency. $\mathrm{Re}[K(\omega)]$ is limited between -0.5 and 1. $|E_{rms}|^2$ is the square of electric field strength in root mean square value.

According to Eq. S1, the DEP force is proportional to the particle radius to the third power. As a result, DEP force becomes the dominant effect among all the AC electrokinetic (ACEK) effects driving forces because of the large particle size of S-protein compared with other particles such as ions. According to previous investigation, the applied voltage to the electrodes is related to the DEP force and the target enrichment. A higher voltage for test is better in theory, but too high a voltage will cause non-specific adsorption, and will be easy to cause adsorption saturation. Therefore, optimization should be performed for the applied voltage.

a

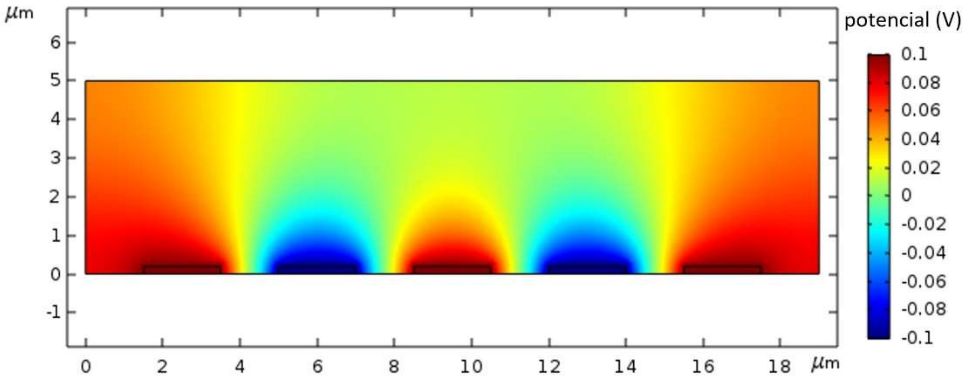

b

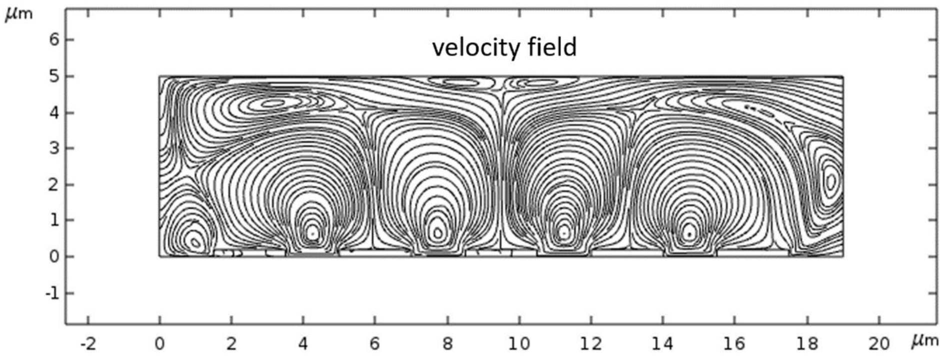

Fig. S3 Field simulation results in the microfluid near the sensor. a potential and **b** velocity field near the IDME surface, where the adjacent electrodes are applied with voltages of reverse phase, and the voltage amplitude is 100 mV with a frequency of 100 kHz.

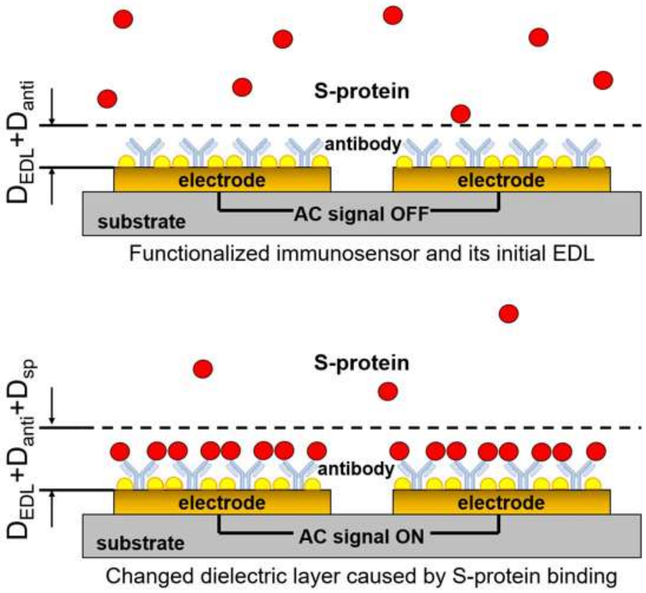

Fig. S4 Diagram for S-protein sensing mechanism. a Random S-protein in the solution and initial dielectric layer before AC signal stimulation. Here the dielectric layer is defined for the complex layer between electrode and homogeneous bulk liquid. This layer composed of EDL and the immobilized or adsorbed dielectric particles works as a dielectric medium for the interfacial capacitor. **b** Adsorbed S-protein on IDME surface and changed dielectric layer after AC signal stimulation. The Y-shaped units are antibodies immobilized on the electrode surface, the rad spheres represent S-proteins, and the yellow particles are lactalbumin blockers. The initial dielectric layer is composed of EDL and antibody layer

(DEDL+ Danti), and the dielectric layer becomes thicker due to S-protein adsorption (DEDL+ Danti+Dsp).

Eq. S2:

$$\frac{\Delta C}{C_{int-b}} = \frac{C_{int-a} \quad -C_{int-bef}}{C_{int-b}} = \left(\frac{A}{\frac{d_{EDL}}{\varepsilon_{EDL}} + \frac{d_{anti}}{\varepsilon_{anti}} + \frac{d_{sp}}{\varepsilon_{sp}}} - \frac{A}{\frac{d_{EDL}}{\varepsilon_{EDL}} + \frac{d_{anti}}{\varepsilon_{anti}}} \right) \div \left(\frac{A}{\frac{d_{EDL}}{\varepsilon_{EDL}} + \frac{d_{anti}}{\varepsilon_{anti}}} \right)$$

$$= \frac{-d_{sp}}{d_{sp} + \frac{\varepsilon_{sp}}{\varepsilon_{EDL}} d_{EDL} + \frac{\varepsilon_{sp}}{\varepsilon_{anti}} d_{anti}} \qquad \text{Eq. S2}$$

In Eq. S2, $C_{int-bef}$ is the initial interfacial capacitance, C_{int-a} is the interfacial capacitance after S-protein adsorption, and $\frac{\Delta C}{C_{int-b}}$ is the normalized capacitance change as a sensitive indicator for S-protein sensing. A is the effective surface of the capacitance, assuming a negligible change before and after target binding. ε is the permittivity, and d represents the thickness of a dielectric layer, both with the subscripts of *EDL*, *anti* and *sp*, representing electric double layer, antibody and S-protein, respectively. According to the final expression in Eq. S2, normalized capacitance change is demonstrated again to be negative.

a

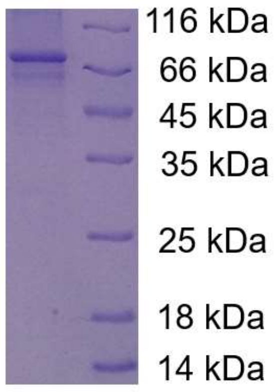

b

MSQCVNLTTRTQLPPAYTNSFTRGVYYPDKVFRSSVLHSTQDLFLP
FFSNVTWFHAIHVSGTNGTKRFDNPVLPFNDGVYFASTEKSNIIRG
WIFGTTLDSKTQSLLIVNNATNVVIKVCEFQFCNDPFLGVYYHKNN
KSWMESEFRVYSSANNCTFEYVSQPFLMDLEGKQGNFKNLREFVF
KNIDGYFKIYSKHTPINLVRDLPQGFSALEPLVDLPIGINITRFQTLLA
LHRSYLTPGDSSSGWTAGAAAYYVGYLQPRTFLLKYNENGTITDAV
DCALDPLSETKCTLKSFTVEKGIYQTSNFRVQPTESIVRFPNITNLCP
FGEVFNATRFASVYAWNRKRISNCVADYSVLYNSASFSTFKCYGVS
PTKLNDLCFTNVYADSFVIRGDEVRQIAPGQTGKIADYNYKLPDDF
TGCVIAWNSNNLDSKVGGNYNYLYRLFRKSNLKPFERDISTEIYQA
GSTPCNGVEGFNCYFPLQSYGFQPTNGVGYQPYRVVVLSFELLHA
PATVCGPKKSTNLVKNKCVNFNFNGLTGTGVLTESNKKFLPFQQFG
RDIADTTDAVRDPQTLEILDITPCSFGGVSVITPGTNTSNQVAVLYQ
DVNCTEVPVAIHADQLTPTWRVYSTGSNVFQTRAGCLIGAEHVN
NSYECDIPIGAGICASYQTQTNSPRRARLEHHHHHH

Fig. S5 Protein information of recombinant S1 subunit. a Stripes from a sodium dodecyl sulfate polyacrylamide gel electrophoresis (SDS-PAGE). **b** The amino acid sequence of the S1 subunit S-protein. The molecular weight is tested to be 75.3 kDa, with a purity above 90%. All the information is provided by Cellregen (Beijing) Life Science and Technology Co., LTD, China.

a b

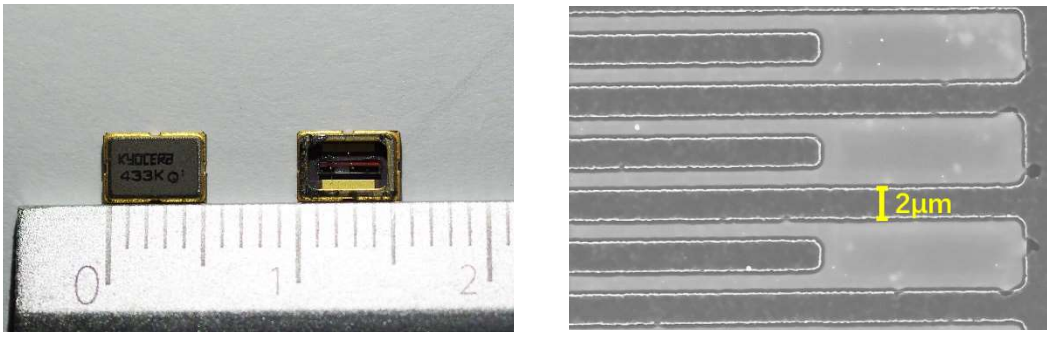

Fig. S6 The interdigitated microelectrode (IDME) chip for sensor preparation. a Encapsulated (left) and cover removed (right) IDME chips with the model number of KYOCERA 433K bought from AVX Corps. **b** The zoom-in scanning electron microscope (SEM) picture of the IDME structure.

a

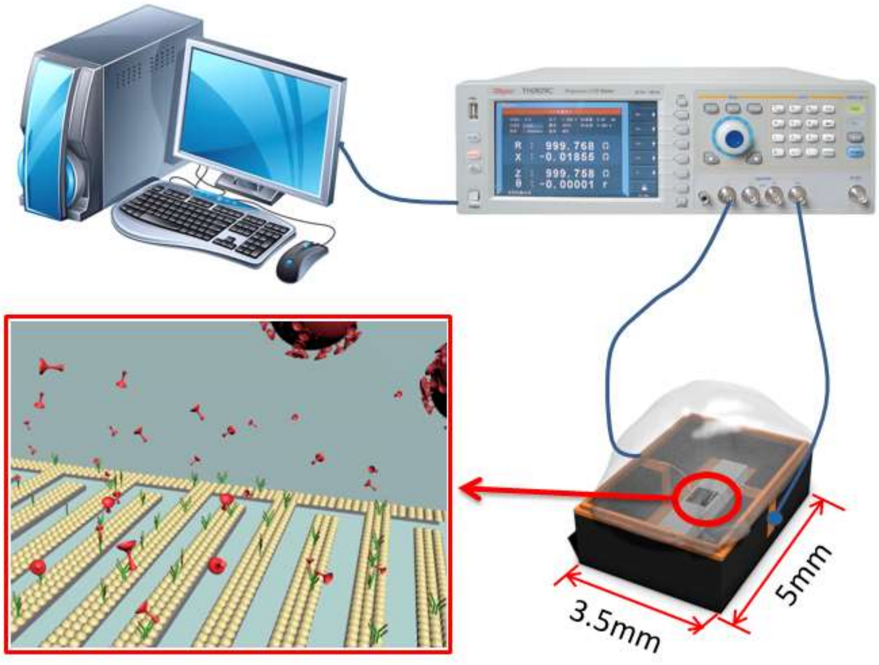

b

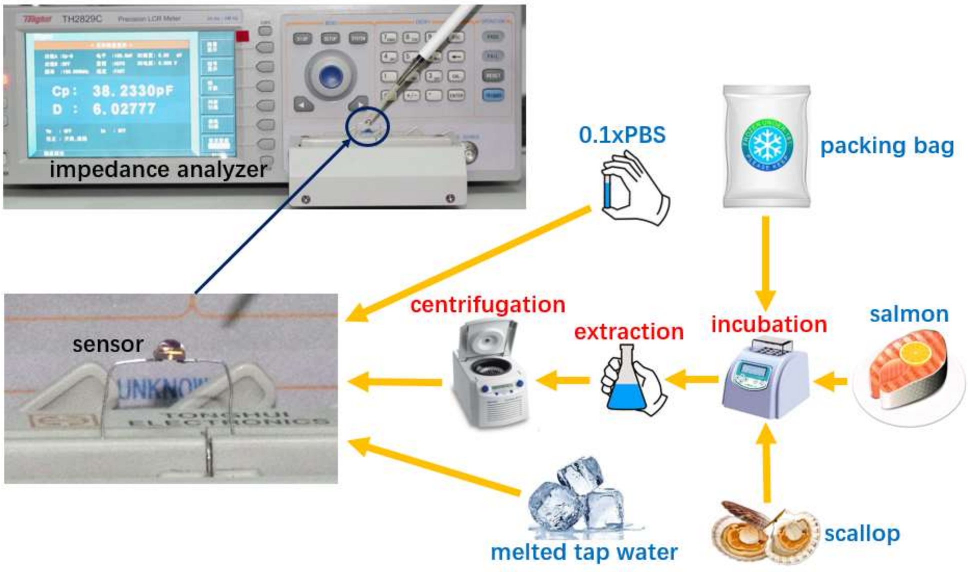

Fig. S7 The measurement system and test procedures. a A schematic of the whole measurement system as well as a diagram for recognition and adsorption of S-protein using the sensor. During the process of enrichment, the interfacial capacitance measurement is integrated, which is executed by an impedance analyzer. **b** Photos of the impedance analyzer and an IDME immunosensor connected to it. The samples and their test procedures involved in this work are visually provided. The immunosensor is disposable, and the whole system is lightweight and cost-efficient for large-scale application in cold-chain food quarantine.

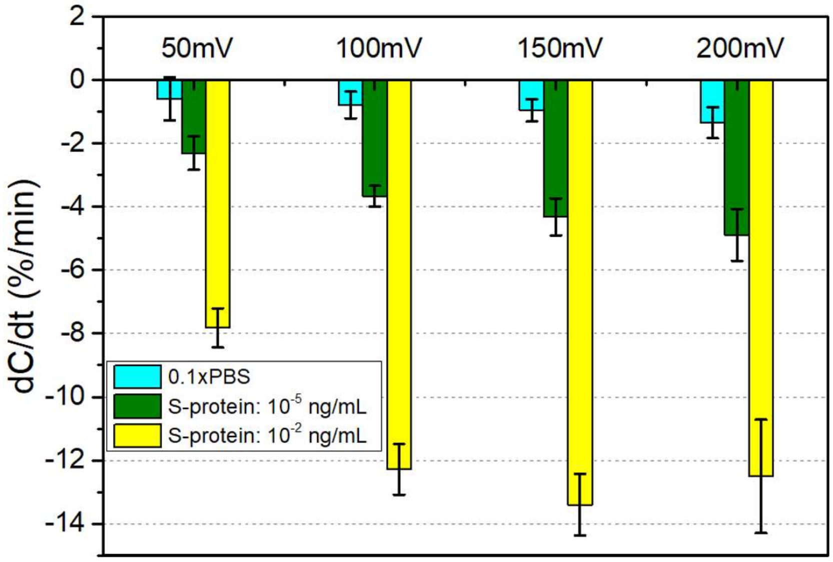

Fig. S8 Voltage optimization for S-protein detection. Two concentrations of 10^{-5} and 10^{-}

^{2} ng/mL are tested, as well as their background of 0.1×PBS solution. The applied voltage is changed from 50 to 200 mV at intervals of 50 mV. When the concentration is 10^{-5} ng/mL, the response increases with higher voltage for all voltages tested. As the concentration rises to 10^{-2} ng/mL, the increase in response slows down at 150 mV. At this concentration, the response even becomes smaller at 200 mV than that at 150mV, indicating an adsorption saturation. In fact, the larger standard deviation also indicates a worse consistency when the voltage rises to 200 mV. Therefore, 100 mV is selected to be the working voltage to ensure sufficient response and minimize adsorption saturation.
